# Dispersal ability and its consequences for population genetic differentiation and diversification

**DOI:** 10.1098/rspb.2022.0489

**Published:** 2022-05-25

**Authors:** Daniel Suárez, Paula Arribas, Eduardo Jiménez-García, Brent C. Emerson

**Affiliations:** ^1^ Island Ecology and Evolution Research Group, CSIC Institute of Natural Products and Agrobiology (IPNA-CSIC), C/Astrofísico Francisco Sánchez 3, La Laguna, Tenerife, Canary Islands 38206, Spain; ^2^ School of Doctoral and Postgraduate Studies, University of La Laguna, 38200 La Laguna, Tenerife, Canary Islands, Spain

**Keywords:** dispersal, population structure, diversification, arthropod, spider, beetle

## Abstract

Dispersal ability is known to influence geographical structuring of genetic variation within species, with a direct relationship between low vagility and population genetic structure, which can potentially give rise to allopatric speciation. However, our general understanding of the relationship between dispersal ability, population differentiation and lineage diversification is limited. To address this issue, we sampled mitochondrial DNA variation within lineages of beetles and spiders across the Canary Islands to explore the relationships between dispersal ability, differentiation within lineages and diversification. We found positive relationships between population genetic structure and diversification for both beetles and spiders. Comparisons between dispersive and non-dispersive lineages revealed significant differences for both lineage differentiation and diversification. For both taxa, non-dispersive lineages had stronger population genetic structure. Genus-level endemic species richness and proxies for diversification rate within genera were higher in non-dispersive taxa for both beetles and spiders. Comparisons of average and maximum node divergences within genera suggest that species turnover may be higher in non-dispersive genera. Our results reveal a model where dispersal limitation may shape the diversity of lineages across evolutionary timescales by positively influencing intraspecific and species diversity, moderated by higher extinction rates compared to more dispersive lineages.

## 1. Introduction

Dispersal ability, defined as the movement of an individual from its natal site to another breeding site *sensu* Clobert *et al*. [1], is known to minimize competition and inbreeding, while also allowing individuals to encounter new patches of habitat for resource exploitation [2]. Dispersal ability thus has major effects on individual fitness, population dynamics and species distributions. Dispersal ability also has important consequences for the geographical structuring of genetic variation within species [3], with a clear link between low vagility and population genetic structure (e.g. [4–7]). For a given patchily distributed species, where patches are defined by suitable habitat, high dispersal ability is expected to favour higher rates of gene flow among patches, thus favouring population cohesion. Over an evolutionary timescale this is expected to suppress speciation events and thus clade level diversification [8,9]. In contrast, low dispersal ability will favour reduced gene flow among patches, which in turn will favour the geographical structuring of genetic variation among them through random mutation and genetic drift [10], potentially driving strong allopatric population differentiation (e.g. [11,12]). However, for speciation to be successful, there are three requisites: population splitting, the evolution of reproductive isolation and the persistence of incipient species [13]. Dispersal limitation is expected to positively influence the first two, by promoting population differentiation, but can have both beneficial or detrimental effects on population persistence [2]. Limited dispersal ability may enhance extinction probability by constraining the colonization of empty habitat patches, or by limiting new recruitment within declining populations [14]. However, dispersal limitation may also limit net loss from populations, particularly if patches are small and isolated and dispersal vectors are high, such as islands where it has been noted that winds can promote such a dynamic [15].

Our general understanding of the relationship between dispersal ability, population differentiation and diversification remains limited. In recent years attempts have been made to explore the relationship between population structuring and diversification. Using a dataset comprising a phylogeny of more than 170 species of New World birds, Harvey *et al*. [16] have revealed population differentiation to be positively related to speciation rate, and suggest that the processes involved in population differentiation are connected to those that promote species diversification. By contrast, an analysis of isolation by distance and population differentiation of 104 Australian lizard species found no evidence for a relationship between population differentiation and species formation [17]. Similarly, Nistchke *et al*. [18] found that rates of population differentiation within Australian sea snakes are not positively related to speciation. Both studies suggest that population differentiation is not the rate-limiting step in species formation and that alternative ecological and historical factors are primary determinants of speciation rates. While it is informative to understand dispersal as a factor driving either speciation and/or population differentiation, there is a lack of studies that simultaneously incorporate all three processes. Here we aim to address this using well-characterized arthropod assemblages within an insular oceanic framework.

Recent evidence has been found for a positive relationship between dispersal limitation in beetles and the geographical structuring of genetic variation. Using a standardized sampling approach, Salces-Castellano *et al*. [19] analysed genetic variation from 214 beetle lineages sampled across a singular cloud forest habitat in an oceanic archipelago setting. This study found that population genetic structure was significantly higher for wingless lineages at both the archipelago and island scales, raising the question of how dispersal ability might further influence diversification. Do the patterns observed by Salces-Castellano *et al*. [19] translate to a model of higher diversification when individual dispersal ability is limited (e.g. [16]), or a model where dispersal ability and diversification are uncoupled (e.g. [17,18])? In addition to providing a useful framework to understand the consequences of dispersal ability for population genetic structuring, oceanic archipelago settings can also be leveraged to understand the relationship between dispersal limitation and diversification. *In situ* diversification can constitute and important driver for the assembly of oceanic biotas, generating endemic monophyletic clades [20,21] providing potential to estimate diversification across multiple independent evolutionary lineages within a common sampling framework. Additionally, reduced dispersal ability is frequently associated with insular biotas (e.g. [15,22–25]), emphasizing the role of oceanic archipelagos as exceptional geographical templates to study the evolutionary consequences of dispersal limitation.

Here we take advantage of the same sampling framework employed by Salces-Castellano *et al*. [19] to explore relationships among dispersal ability, differentiation within species, and diversification, while also extending sampling to spider assemblages. Both arthropod orders are appropriate candidates to explore these relationships because: (i) both harbour a sufficient number of species for a comparative approach, with 317 and 1314 endemic species in the Canary islands for spiders and beetles, respectively (Biodiversity Data Bank of the Canary Islands, https://www.biodiversidadcanarias.es/biota/); (ii) both orders are comparatively well understood taxonomically within the Canary Islands, including a well-established characterization of species as either endemic or non-endemic within the archipelago; and (iii) both orders can be partitioned into poor and good dispersers using well-defined traits linked to dispersal potential, i.e. wing development in beetles [19,26] and ballooning ecology in spiders [27–29].

We first test if the geographical structuring of genetic variation within spiders, both between and within islands, is higher for non-dispersive species. To achieve this, we use the same spatial setting previously used to assess geographical structuring of genetic variation within beetles, and a comparable definition of lineages of maternal dispersal history (LMDH) [19], by applying a conservative maximum intraspecific divergence threshold for spiders [30]. Linear regressions between geographical structuring within LMDHs and species number within genera are performed for both orders, to test for a relationship between dispersal ability and diversification at the island scale. Finally, within each order, we add publicly available sequence data to analyse variation among endemic species within genera to test if dispersal ability is associated with diversification at the archipelago scale, comparing endemic species richness, mean node divergence, and maximum node divergence and diversification rate per genus. We predict that: (i) similar to beetles, non-dispersive spider LMDHs will present higher levels of population differentiation; (ii) dispersal limitation will lead to higher diversification, through higher net diversification rates in both orders; and (iii) geographic structuring of genetic variation will be directly correlated with diversification rate within genera.

## 2. Material and methods

### (a) Field sampling

**Figure 1.**
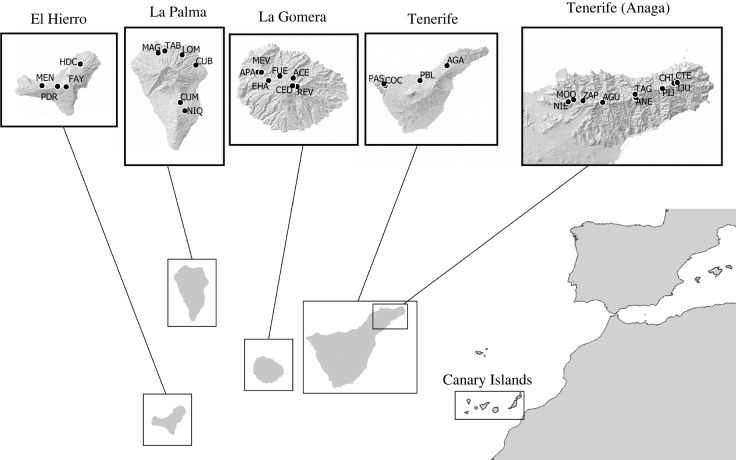
Sampling sites within the laurel forests of the Canary Islands. Sampling sites are labelled with three letter codes (see electronic supplementary material, table S1 for further details).

A total of 31 sites of 50 × 50 m were sampled within laurel forests across the four western islands of the Canary archipelago: Tenerife (14), La Gomera (7), La Palma (6) and El Hierro (4) (figure 1). At each site, a standardized protocol combining passive sampling (pitfall traps) and active sampling techniques (foliage beating, vegetation sweeping, active searching and leaf litter-sifting) was applied (see [30] for further details). Sampling was carried out from 2012 to 2020 between the months of November and May. Samples were preserved in absolute ethanol at −20°C until further examination. Six sites (two in each of Tenerife, La Palma and El Hierro) were not included in Salces-Castellano *et al*. [19], and thus for comparative purposes, they were also sampled for their beetle fauna, sequenced and processed, as described in Salces-Castellano *et al*. [19].

### (b) Mitochondrial DNA sequencing

Samples were classified into parataxonomic units (PU) by direct examination of external morphology under a binocular lens. Up to four individuals per PU per site were selected for DNA extraction and sequencing. Depending upon the specimen size, a single leg, several legs or prosome were digested using a Chelex protocol [31]. The 5′ region (658 bp) of the mtDNA COI gene was amplified using the LCO1490 and HCO2198 primers [32]. PCR reaction conditions were as follows: initial denaturation at 94°C for 2 min, followed by 40 cycles of 94°C for 30 s, 42–46°C for 35 s and 72°C for 45 s, and a final extension of 72°C for 5 min. Diluted (1/10) DNA extract (1–2 µl) was amplified with 24–23 µl of PCR mix (for a total volume of 25 µl) comprised of 14.4 µl water, 2.5 µl of 10× NH_4_ buffer (Bioline), 1.5 µl of 50 mM MgCl_2_ (Bioline), 2 µl of 2.5 mM dNTPs (Bioline), 0.5 µl of BSA (20 mg ml^−1^), 1 µl of each primer (10 µM) and 0.1 µl of Taq polymerase (BIOTAQ). PCR products were sequenced using the Sanger DNA sequencing service of Macrogen (www.macrogen.com) with either the forward or reverse primer or both primers in the event of insufficient read length from a single primer. Sequences were then edited in Geneious v. 2021.1.1 (www.geneious.com).

### (c) Mitochondrial DNA lineage delimitation for the estimation of dispersal history

To harmonize data with that of Salces-Castellano *et al*. [19], we adopted their approach by defining LMDHs, that minimize the probability of a given biological species being assigned to more than one LMDH, while simultaneously providing a similar time frame for comparisons among lineages. While mtDNA substitution rate variation may be expected across different spider lineages [33], there is no evidence that these should correlate with species dispersal ability. A custom R script [19] was used to produce an unweighted pair group method with arithmetic mean (UPGMA) tree from pairwise K2P distances using an alignment of all sequences from all PUs. A conservative maximum intraspecific divergence threshold of 6.8% was used, above which it is unlikely for individuals from the same biological species to be assigned to more than one spider LMDH [30,34]. LMDHs may thus represent different stages of the speciation process, from single panmictic species, through to geographically structured species, incipient species, and species complexes, and ultimately different taxonomic species, which may or may not be taxonomically diagnosable. Each LMDH was taxonomically assigned to species or genus level and categorized for dispersal ability. The potential for juvenile spider stages to be passively dispersed by air currents, while suspended from silk threads, henceforth referred to as ‘ballooning’, was considered a proxy for dispersal ability [27,28]. LMDHs were categorized as either ballooning or non-ballooning following a family-level classification established by Carvalho & Cardoso [29].

### (d) Geographical structuring of genetic variation at the LMDH level

To test for genetic structure within LMDHs, two indices of fixation were generated: *G*_ST_ (genetic distance among haplotypes is unweighted) and *N*_ST_ (genetic distance among haplotypes is weighted). LMDHs sampled from a minimum of two populations and comprising more than three individuals were selected to estimate *G*_ST_ and *N*_ST_, both within individual islands (considering each sampling site as a population) and among islands (considering each island as a population) using SPAGeDi 1.5 [35].

### (e) Diversification proxies at the genus level

The mean number of endemic species within genera at the archipelago scale was compared between dispersive and non-dispersive lineages. Data on endemic species richness per genus were extracted from the Biodiversity Data Bank of the Canary Islands (https://www.biodiversidadcanarias.es/biota/, accessed January 2021). For phylogenetic measures, genus-level alignments were generated using single sequences from each species sampled in this study, and additional sequences available on BOLD or GenBank. For beetles, in addition to the region sequenced by Salces-Castellano *et al*. [19], we also generated alignments for the barcode region. Thus, two alignment regions were used, COIa (the 658 bp barcode region) for spiders and beetles, and COIb (a non-overlapping downstream region of 735 bp) for beetles.

Endemic species richness and measures of phylogenetic divergence were used to derive proxies for diversification at the genus level. Specifically, we used (i) endemic species number within each genus and (ii) endemic species number divided by maximum node divergence within a genus as a proxy measure of diversification rate, corresponding to age-richness rate estimators (ARR). We also explored the average node divergence within genera (the average of the individual divergence estimates associated with each node) and the maximum node divergence within genera (a proxy for the crown age) to explore the temporal context of lineage diversification. There are recognized theoretical issues associated with the use of ARR estimators [36], however, these should be less consequential when comparing across groups of independently sampled lineages, for which all speciation events are relatively close to the present. We sought to minimize overestimating diversification rates by subdividing non-monophyletic genera within the archipelago (i.e. species are derived from more than one colonization event) into alignments corresponding to each colonization event. Also, if a given genus comprised more than one subgenus with mainland relatives, subgenera were similarly analysed independently.

Mean and maximum node divergences were estimated independently for each alignment using two different tree-based approaches, UPGMA with uncorrected *p*-distances and Bayesian estimation. For UPGMA trees, node divergences were estimated using the function ‘tree.age’ from the package dispRity [37]. Node divergences from Bayesian trees were estimated with BEAST v. 2.6.4. [38], applying (i) a Kimura two-parameter substitution model for each alignment, (ii) a relaxed lognormal molecular clock, (iii) a birth–death tree prior and (iv) a normal prior distribution representing the 95% confidence interval around specific rates of 0.0125 (±0.0036) for mtDNA COIa in spiders [39], 0.0168 (±0.0018) and 0.0177 (±0.0019) for COIa and COIb respectively in Polyphaga [40], and 0.0113 (±0.0034) and 0.0145 (±0.0054) for COIa and COIb respectively in Adephaga [41]. MCMC analyses were run for 100 000 000 steps, sampling every 1000 steps, with the first 25% discarded as burn-in, using values of effective sample size (ESS) greater than 200 as a minimum for acceptance. Both approaches were run independently for each genus-level alignment.

### (f) Relationship among dispersal ability, genetic differentiation and diversification

To test for a relationship between genetic structuring and diversification across all dispersive and non-dispersive lineages within each order, Spearman rank correlation tests (cor.test function, stats R package) were applied to compare mean *G*_ST_ and *N*_ST_ indexes for all the species within a genus, with the corresponding endemic species richness, both within individual islands (island scale) and among islands (archipelago scale). To test for a relationship between dispersal ability and the geographical structuring of genetic variation at both archipelago and island scales, fixation indices were compared between dispersive and non-dispersive lineages, for both spiders and beetles, using Wilcoxon rank-sum tests in R v. 4.0.4 (wilcox.test function, stats R package). At the island scale, a linear mixed-effect model was constructed using the lmer function (lme4 package) [42], implementing ‘*N*_ST_’ as the response variable and ‘dispersal ability’ as the predictor variable, and including ‘island’ as a random intercept as well as a random slope. Conditional modes of the random effects, i.e. differences between intercept and slope for each island and the overall intercept and slope, were extracted using the ranef function (lme4 package) [42]. Finally, to test for a relationship between dispersal ability and genus-level diversification, species number, mean node divergence, maximum node divergence and diversification rate were compared between dispersive and non-dispersive genera using Wilcoxon rank-sum tests. Additionally, to take into account potential non-independence of transitions from the dispersive to non-dispersive state, a phylogenetic least squares (PGLS) analysis was conducted using the gls function (nlme package) [43]. Neighbour-joining phylogenetic trees were generated using all available COIa and COIb sequences for spiders and beetles respectively, and then pruned to contain one species per genus.

## 3. Results

### (a) Field sampling and DNA sequencing

A total of 21 082 spider individuals were collected and classified into 148 PUs, from which 3338 individuals were selected for sequencing, yielding a total of 2663 sequences, representing a sequencing success rate of 79.7%. Across the six sites newly sampled for their beetle fauna, a total of 4266 specimens were collected, from which 1817 were selected for sequencing, yielding a total of 1594 sequences, representing a sequencing success rate of 87.7%. Incorporating beetle data from Salces-Castellano *et al*. [19] yielded a total of 333 LMDH, 214 for beetles and 119 for spiders. Within spiders, 55 lineages (46.2%) were classified as having limited dispersal ability (i.e. non-ballooner), with the remaining 64 having high dispersal ability (i.e. ballooner). Within beetles, 110 lineages (51.4%) were classified as having limited dispersal ability (i.e. wingless), with the remaining 104 classified as having high dispersal ability (i.e. winged). At the island scale, the total number of LMDHs recovered were 234 for Tenerife (80 spider and 154 beetle), 168 for La Gomera (62 spider and 106 beetle), 150 for La Palma (49 spider and 101 beetle) and 111 for El Hierro (45 spider and 66 beetle). Among these, fixation indices could be calculated for 100 LMDHs in Tenerife (33 spider and 67 beetle), 65 LMDHs in La Gomera (28 spider and 37 beetle), 49 LMDHs in La Palma (21 spider and 28 beetle) and 33 LMDHs in El Hierro (14 spider and 19 beetle). LMDHs may include more than one taxonomic species, particularly within evolutionary lineages that have experienced recent speciation. Across both orders there were only 42 cases (three spider and 39 beetle) from a total of 333 where a LMDH comprised more than one taxonomic species. Thirty of the 42 cases comprised two taxonomic species occurring on different islands.

### (b) Dispersal limitation and population genetic differentiation

**Figure 2.**
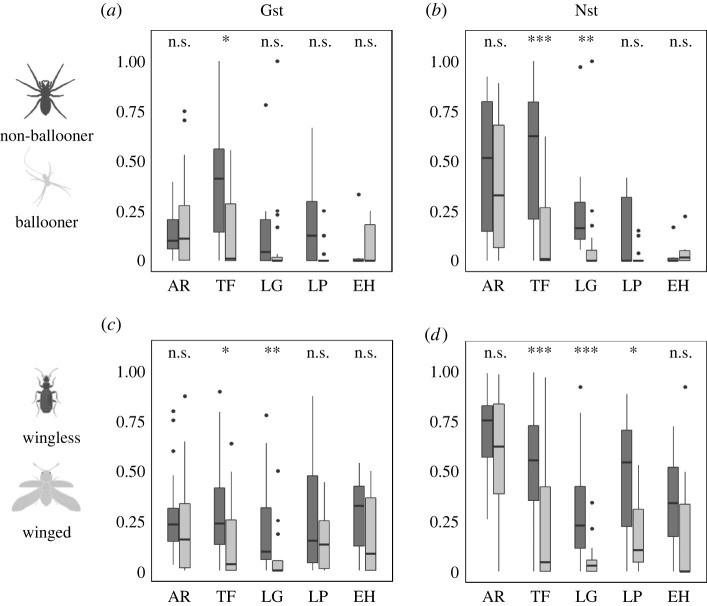
Fixation indices for non-dispersive (dark grey) and dispersive (light grey) lineages. (*a*) *G*_ST_ and (*b*) *N*_ST_ for spider lineages. (*c*) *G*_ST_ and (*d*) *N*_ST_ for beetle lineages. AR = archipelago, TF = Tenerife, LG = La Gomera, LP = La Palma, and EH = El Hierro. ***p < 0.001, **p < 0.01, *p < 0.05, n.s. = not significant.

Linear mixed-effects models using *N*_ST_ showed statistically significant differences between dispersive and non-dispersive beetles (*p* = 0.02) but not between dispersive and non-dispersive spiders (*p* = 0.13). For spiders, conditional modes of the random effect in El Hierro and La Palma were lower than the general model (El Hierro: intercept = −0.05, slope = −0.13; La Palma: intercept = −0.03, slope = −0.07) while in La Gomera and Tenerife they were higher (La Gomera: intercept = 0.01, slope = 0.15; Tenerife: intercept = 0.07, slope = 0.19). For beetles, conditional modes of the random effect in El Hierro and La Gomera were lower than the general model (El Hierro: intercept = −0.01, slope = −0.003; La Gomera: intercept = −0.08, slope = −0.02) while in La Palma and Tenerife they were higher (La Palma: intercept = 0.02, slope = 0.01; Tenerife: intercept = 0.06, slope = 0.02). Wilcoxon rank-sum tests revealed that there was no significant difference between dispersive and non-dispersive lineages, neither for spiders (*p* = 0.27, *n* = 44) nor beetles (*p* = 0.11, *n* = 66), when comparing *N*_ST_ at the scale of the entire archipelago. At the scale of individual islands, significant differences were found between dispersive and non-dispersive beetle lineages on the islands of Tenerife (*p* = 0.003, *n* = 68), La Gomera (*p* = 0.0004, *n* = 37) and La Palma (*p* = 0.03, *n* = 29), and between dispersive and non-dispersive spider lineages on the islands of Tenerife (*p* = 0.004, *n* = 33) and La Gomera (*p* = 0.002, *n* = 28). Results for both *N*_ST_ and *G*_ST_ are presented in figure 2.

### (c) Geographical structuring of genetic variation and diversification

For *N*_ST_ at the archipelago scale, all analyses for both orders revealed a positive relationship (electronic supplementary material, figure S1). Significant relationships were found for the combined analysis of all dispersive and non-dispersive spider genera (*n* = 31, *p* = 0.0006), as well as non-dispersive genera alone (*n* = 13, *p* = 0.03), while the relationship was not significant for dispersive genera (*n* = 18, *p* = 0.24). For beetle genera, no significant relationships were found for any of the three analyses. Analyses using *G*_ST_ followed a similar positive trend, with a significant relationship found for the combined analysis of all dispersive and non-dispersive spider genera (*n* = 31, *p* = 0.006), while the relationship for non-dispersive genera alone was only marginally significant (*n* = 13, *p* = 0.07) and for dispersive genera it was not significant (*n* = 18, *p* = 0.51) (electronic supplementary material, figure S2). At the scale of individual islands, significant relationships were found for spiders within Tenerife for the combined analysis of all dispersive and non-dispersive genera (*n* = 23, *p* = 0.002) as well as individually (dispersive, *n* = 13, *p* = 0.02; non-dispersive, *n* = 10, *p* = 0.09) (electronic supplementary material, figure S3). Significant relationships were found in La Gomera for non-dispersive spider genera (*n* = 7, *p* = 0.02) (electronic supplementary material, figure S4). Significant relationships were also found in La Palma for dispersive spider genera (*n* = 10, *p* = 0.02) (electronic supplementary material, figure S5). Although there was a tendency toward a positive relationship for beetle genera at the scale of individual islands, no relationships were significant (electronic supplementary material, figure S3–6). Relationships between *G*_ST_ and species richness within islands were similar to those found with *N*_ST_ (electronic supplementary material, figures S7–10).

### (d) Dispersal limitation and genus level diversification

**Figure 3.**
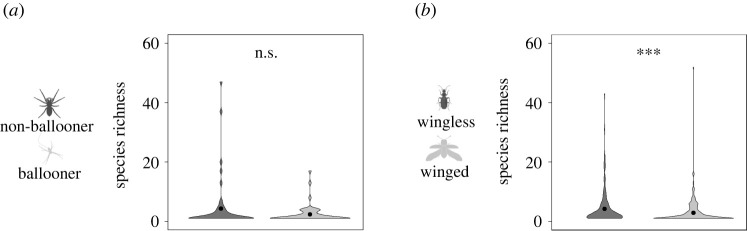
Violin plot of species number per genera for non-dispersive (dark grey) and dispersive (light grey) lineages for spiders (*a*) and beetles (*b*). Plot width represents the relative number of genera with a given species richness. ***p < 0.001, **p < 0.01, *p < 0.05, n.s. = not significant. For aesthetic purposes, an outlier wingless beetle genus (*Laparocerus*) with 173 species was omitted.

While all spider genera could be successfully characterized for their dispersal ability, a lack of robust information on the presence or absence of wings for many beetle genera meant that only 335 of the 416 genera (80.5%) were assigned to a dispersal category. Wilcoxon rank-sum tests revealed that species richness within spider genera was not significantly different between dispersive (*n* = 92, mean richness = 2.35) and non-dispersive (*n* = 76, mean richness = 4.38) genera (*p* = 0.41). By contrast, species richness was significantly different (*p* = 0.0007) between dispersive (*n* = 198, mean richness = 2.93) and non-dispersive (*n* = 137, mean richness = 5.65) beetle genera (figure 3). The beetle comparison was repeated removing an outlier (genus *Laparocerus*, richness = 173), with the difference remaining significant (*p* = 0.001). The results also remained significant when accounting for potential phylogenetic covariance. For the PGLS analyses, a total of 519 sequences were retrieved (195 for spiders and 304 for beetles) belonging to 167 genera (62 spider and 105 beetle). Analyses revealed that species richness was significantly different (*p* = 0.04) between dispersive and non-dispersive beetle genera while for between dispersive and non-dispersive spider genera the difference was non-significant (*p* = 0.20).

**Figure 4.**
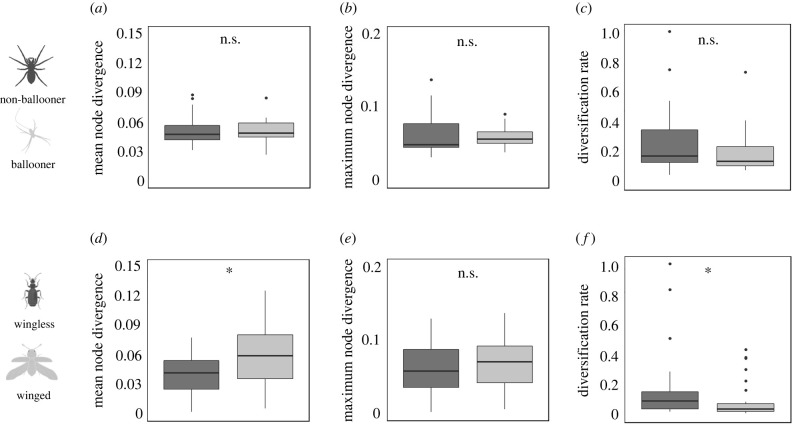
Box plots of mean and maximum node divergences, estimated using BEAST, and diversification rate estimates for non-dispersive (dark grey) and dispersive (light grey) lineages. (*a*) Mean node divergences within spider genera. (*b*) Maximum node divergences within spider genera. (*c*) Proxy for diversification rate within spider genera, normalized from 0–1. (*d*) Mean node divergences within beetle genera. (*e*) Maximum node divergences within beetle genera. (*f*) Proxy for diversification rate within beetle genera, normalized from 0–1. ***p < 0.001, **p < 0.01, *p < 0.05, n.s. = not significant.

For diversification analyses, only genera that could be assigned to a dispersive category, and that comprised two or more species with DNA sequence data were considered. Thus, UPGMA and Bayesian gene trees were estimated for a total of 655 species (153 spider and 502 beetle) from 99 genera (29 spiders and 70 beetles, electronic supplementary material, table S2). Taking into account the total number of endemic species across the archipelago for both orders (317 spiders and 1314 beetle) and total number of genera comprising two or more endemic species (47 spiders and 198 beetle), the percentage representation for species richness was 40% (48% for spiders and 38% for beetles) while for number of genera it was 41% (62% for spiders and 35% for beetles). Analyses using node divergences estimated from UPGMA and Bayesian trees yielded similar results, with only minor differences between them. We present results derived from Bayesian trees (figure 4), with results from UPGMA trees presented in the electronic supplementary material (electronic supplementary material figure S11). Non-dispersive beetle genera were found to have significantly higher diversification rates compared to dispersive genera (*p* = 0.01). Similarly, the mean diversification rate for non-dispersive spider genera was higher than that of dispersive genera, but the difference was not significant (*p* = 0.37). Mean node divergences were significantly younger in non-dispersive beetle genera, compared to dispersive genera (*p* = 0.02). While the difference between dispersive and non-dispersive spider genera trended in the same direction, the difference was not significant (*p* = 0.98). There were no significant differences for maximum node divergence between dispersive and non-dispersive genera in either order (spiders, *p* = 0.60; beetles, *p* = 0.42).

## 4. Discussion

### (a) Dispersal limitation as a driver of population differentiation and diversification

Positive linear relationships were found between differentiation and diversification both at archipelago and island scales (electronic supplementary material, figures S1–10), being significant for spiders but not for beetles. Within the latter, there are many data points with relatively high *N*_ST_, but with richness values close to 1, which is particularly apparent for wingless beetle genera. This points to the presence of genera with few species, but where species experience high geographical structuring of genetic variation. Why this genetic structuring is not reflected in higher species richness could be related to either (i) high extinction rates within these genera, or (ii) a recent origin of such genera within the archipelago. Overall, the positive relationships observed contrast with the findings of Singhal *et al*. [17] and Nitschke *et al*. [18], but are consistent with Harvey *et al*. [16]. However, none of these previous studies incorporate the role of dispersal limitation in their models. Dispersal limitation can lead to the limited or complete absence of gene flow among populations over evolutionary timescales, providing conditions that should favour geographical structuring of genetic variation within species, and eventually speciation. Consistent with this, we find that population genetic structure in spiders and in beetles tends to be higher in non-dispersive lineages, at both archipelago and island scales, in line with similar findings from Salces-Castellano *et al*. [19]. Linear mixed models revealed a significant tendency for beetles but not for spiders. Thus, the relationship between population genetic structure and dispersal ability for spiders is not general to all islands, potentially reflecting influences of differing island sizes and ages.

For both taxa, *N*_ST_ boxplots (figure 2) reveal an apparent stronger effect of dispersal limitation within islands than among them. While differences were not statistically significant at the archipelago scale, strong statistical differences were found at the within island scale, particularly on older islands. This suggests that the evolutionary consequences of dispersal ability are scale dependent, with species of differing dispersal ability being affected more similarly as the spatial scale of a dispersal barrier increases. However, this interpretation should be taken with caution as (i) each island-scale dataset contains a different set of species, (ii) the archipelago scale dataset is reduced to include only species occurring on more than one island and (iii) due to the low number of non-dispersive species shared across more than one island, the comparison between dispersive categories may be unbalanced. If geographical structuring were to be influenced by those LMDHs comprising more than one taxonomic species, we would expect an effect at the archipelago scale, as non-dispersive lineages tend to have more endemic species restricted to one island than dispersive ones. As *N*_ST_ at the within-island scale is calculated using only genetic data within the island, there is limited impact for bias resulting from LMDHs that include more than one species.

As well as exhibiting similar trends for dispersal ability and geographical structuring of genetic variation, both taxa presented similar trends for the relationship between dispersal ability and diversification. Among beetles, the number of species in a genus is significantly higher for non-dispersive compared to dispersive genera (figure 3), and diversification rates are also significantly higher in non-dispersive genera (figure 4). Estimates for the average mean node divergence were also significantly younger within non-dispersive beetle genera compared to dispersive genera, but with no significant difference between both groups for the maximum node divergence (figure 4). This pattern is suggestive of a model where speciation and extinction rate are both typically higher in non-dispersive genera, but more so for speciation rate, contributing to both higher species richness and species turnover compared to dispersive genera. However, as we did not account for the relative impact of extinction rate in our analysis, this interpretation should be treated with caution. Our results are consistent with Ikeda *et al*. [26], who found that flightless beetle lineages had higher speciation rates than flighted ones, thus placing dispersal limitation as a driver of species richness. In response to Ikeda *et al*. [26], Vogler & Timmermans [44] suggested that loss of flight may be an indirect response to different habitat conditions, thus obviating its role as the driving agent of speciation. By explicitly sampling species from the same habitat and geographical setting, our results strengthen the argument for dispersal limitation's primary role in population differentiation and species formation.

For spiders, results point to similar trends as found for beetles, but there were no significant differences among dispersive and non-dispersive genera for species richness, mean node divergence or diversification rate (figure 4). These results, while pointing to a common model between both groups, suggest that dispersal limitation, as estimated by the absence of ballooning ecology in spiders, is less consequential as a pathway to diversification than the absence of wings in beetles. Immature beetle stages are typically immobile and often hidden within soil or plant tissue, and are thus unlikely to be dispersed by wind. By contrast, immature spiders are much more mobile and can be found on leaf surfaces, thus facilitating passive dispersal by wind, even in the case of non-dispersive species. However, we cannot rule out that misclassification of species-specific ballooning ecologies may contribute to a lack of relationship between dispersal ability and diversification in spiders. In contrast to beetles, where dispersal categorization is made by direct species examination, ballooning categorization is taxonomically inferred at the family level [45]. Island taxa often experience niche shifts compared to their mainland relatives, including loss of dispersal power [46]. Although confamilial spider species tend to have similar life histories [45], we cannot rule out that some species categorized *a priori* as good dispersers may have lost their ability to disperse by ballooning.

### (b) Analytical challenges for extrapolating from differentiation to diversification

Singhal *et al*. [17] have put forward several factors that they suggest may bias analyses of diversification and differentiation, including species delimitation, errors in estimating diversification rate and inappropriate phylogenetic scale. Regarding species delimitation, spiders and beetles are among the more well-studied and understood arthropod groups within the Canary Islands, having been the subject of substantial taxonomic effort over the last two centuries. Thus, the issue of bias in species delimitation is unlikely to be consequential for the estimation of species richness per genera. For diversification rates, using lineages based on an objective genetic divergence threshold instead of species minimizes potential errors associated with taxonomic biases. Errors associated with the estimation of diversification rates may also confound results. The estimation of diversification rates, and its decomposition to speciation and extinction, is increasingly recognized as complex, both for age-richness rate estimates and estimates derived from phylogenetic trees (e.g. [36,47]). Rather than seeking to understand diversification within individual lineages, we instead compare age-richness rate and phylogeny derived inferences for speciation and diversification rates between large groups of independent genera. However, it remains possible that issues described by Louca & Pennell [47] and Rabosky & Benson [36] may weaken the power of our analyses to detect differences.

In the absence of published phylogenies for the great majority of genera, we estimated node divergences among those species for which phylogenetic data were available. Although incomplete sampling may bias node divergence values [48], it is not unreasonable to assume that any such bias should be equally distributed between dispersive and non-dispersive genera. Another potential source of error is the assumption that all endemic species within a genus are monophyletic. Although the most common pattern within the Canary archipelago is single colonization and diversification *in situ* within genera [49], there are examples of multiple colonization's within the same genus (e.g. [50–52]). We have taken account of this by using subgenus affiliation with the continent to partition genera when necessary. Additionally, when a published phylogeny revealed more than one colonization event within a genus, we partitioned the data accordingly. By adopting these measures, we limit the potential for overestimating MRCA node divergences.

Finally, Singhal *et al*. [17] suggest that phylogenetic scale may affect the power to detect relationships between population differentiation and diversification, with broader phylogenetic scales having greater power to detect weaker relationships. Both Singhal *et al*. [17] and Nitschke *et al*. [18] focused on single clades of skinks and sea snakes respectively, at subfamily level, and found no evidence for a relationship. In contrast, Harvey *et al*. [16], recovered a significant relationship between genetic structure and speciation rate at a broader phylogenetic scale comprising a complete phylogeny of Neotropical birds. While our phylogenetic analyses are below the genus level, and thus more recent than that of Harvey *et al*. [16], the power issue raised by Singhal *et al*. [17] is likely to be compensated for by our comparative analytical framework.

## 5. Conclusion

Overall, our results are consistent with a model where population differentiation and diversification are positively associated, which has previously been suggested for New World birds [16]. Our results also point to dispersal limitation as a key factor in both population differentiation and diversification rate. We have found dispersal limitation to be associated with both higher population differentiation and higher diversification rate. Patterns within beetles also suggest that species turnover (species extinction and replacement) may be higher in non-dispersive genera. This argues for higher diversification rates in non-dispersive genera not simply being driven by a higher speciation rate, but also moderated by higher extinction rates than dispersive genera. This is consistent with Brown & Kodric-Brown [14], who suggest that species that disperse poorly are more prone to extinction than dispersive species as, in the latter, local population extinctions can be buffered by dispersers moving into declining populations. Further research is needed to understand how dispersal ability and population turnover through time interact to lead to species formation. However, our results point to a potentially general model where dispersal limitation leads to higher geographical structuring of genetic variation within species, but where diversification rate depends upon both dispersal ability and extinction rate.

## Data Availability

The information associated with the studied lineages, including taxonomic identification (family level identification), dispersal ability assignment, sampling and associated DNA sequences as well as DNA sequences used for diversification analyses, are available from the Dryad Digital Repository [53]. The custom R script developed to define lineages of maternal dispersal history (LMDH) by applying a maximum intraspecific divergence threshold is available from GitHub (https://github.com/asalcescastellano/Divergence-threshold.git). All supplementary tables and figures cited in the main text have been uploaded as electronic supplementary material [54].
